# Women in Orthopaedics: A Perspective from Malaysian Female Orthopaedic Surgeons

**DOI:** 10.5704/MOJ.2303.009

**Published:** 2023-03

**Authors:** SK Liew, JA Lee, F Tamam, II Ismail, F Mohamed-Saaid, PC Chye

**Affiliations:** 1 Department of Orthopaedic Surgery, University Putra Malaysia, Serdang, Malaysia; 2 Department of Orthopaedic Surgery, University Putra Malaysia Teaching Hospital, Serdang, Malaysia; 3 Department of Orthopaedics and Traumatology, Hospital Kuala Lumpur, Kuala Lumpur, Malaysia

**Keywords:** gender, orthopaedics, female surgeons, women, diversity and inclusion

## Abstract

**Introduction:**

The awareness of under-representation of female surgeons in orthopaedics has been increasing in this decade. We aim to investigate the reasons why female surgeons chose orthopaedic, the barriers that possibly hinder female surgeons into orthopaedics and analyse the obstacles that they encountered in their career in Malaysian context.

**Materials and methods:**

A total of 101 registered female orthopaedic surgeons registered with the Malaysian Medical Council, during the period 1980 to 2020, were contacted for a cross-sectional survey, consisting of thirty-four questions on their experience in the orthopaedic career. Eighty-two responses were received (81.2%). Questions in this survey consisted of four sections: (1) demographic details, (2) current clinical practice environment, (3) orthopaedics training experience, and (4) career experience.

**Results:**

A total of 49% of respondents had subspeciality training, highest in paediatric orthopaedic (30%). Enjoyment of manual tasks (64.6%) and professional satisfaction (64.6%) were the top reasons for choosing orthopaedic as a career. Primary barriers to orthopaedic were physical strength required (56.0%) and public gender bias (52.4%). Twenty-eight percent reported gender discrimination in career opportunities while 60% reported similar in daily work. Thirty-three percent reported verbal and 11% physical sexual harassment in their career. Forty-four percent of respondents reported benefits as female orthopaedic surgeon in their work.

**Conclusion:**

The reasons for Malaysian female orthopaedic surgeons to choose orthopaedic as their career and the barriers perceived to hinder other females from choosing orthopaedics were similar to reports worldwide with no exception to gender discrimination and sexual harassment. The support given by male colleagues to married female surgeons marked a unique phenomenon in Malaysian culture.

## Introduction

Orthopaedic surgery has been known to be a male dominated speciality since it was first introduced. Although there has been an increase of female students in medical schools, the percentage of them pursuing an orthopaedic career has been low comparing to other specialties. From a latest report in 2020 on female orthopaedic surgeons’ percentage in global 31 countries, the highest proportion of female surgeons was in Estonia (26.4%)^1^. For Southeast Asia, the highest was in Brunei (13.3%), followed by Malaysia (10%) and Indonesia (5.4%), Thailand (3.8%), The Philippines (3.3%), Singapore (3.2%), Myanmar (2.0%), Cambodia (0%) and Laos (0%)^1^. This trend has been the same even in the developed countries like the United States (6.1%), United Kingdom (4.8%), Sweden (16.8%) and France (7.1%)^1.^

In Malaysia, the percentage of females in public universities far outnumbered males^2^. According to Malaysia’s Gender Gap Index report, the combined gross enrolment ratio was in favour of men in 1980 (53%-56.9%), but parity was achieved by 1990; women have had a higher enrolment ratio since 2000 of 65.3%-64.3%^3^. With only 10% of female orthopaedic surgeons in the country, this reflects those female medical graduates were less likely to choose orthopaedic surgery compared to other specialties.

Female under-representation in orthopaedic surgery in Southeast Asia is alarming and some countries have no female orthopaedic surgeon at all. It is important to analyse the barriers for women in pursuing orthopaedics and change the male-dominant field to a well-balanced environment to serve the diversity of population worldwide. Numerous studies and articles have been published concerning this issue but mostly in the developed countries where the winds of diversity and gender equality are strong. To our best knowledge, there is yet such study conducted in Southeast Asia. We aim to investigate and analyse the reasons of why female orthopaedic surgeons chose orthopaedics as their career, the barriers that could possibly hinders other females from pursuing orthopaedics and the obstacles that they encountered in their career.

## Materials and Methods

This is a cross-sectional qualitative study involving a 34-questions questionnaire survey from 1st October to 30th November 2020. The respondents were female orthopaedic surgeons who were gazetted and registered with the Malaysian Medical Council from the year 1980 to 2020 (n=101). All qualified surgeons were contacted though emails and phones and the survey were answered through an electronic (online) questionnaire. Questions in this survey consist of four sections: (1) demographic details, (2) current clinical practice environment, (3) their orthopaedics training experience, and (4) their career experience as an orthopaedic surgeon.

In Section 2, they were asked about their current status of employment, academic status, subspecialty qualification, current practicing field and reasons of did not pursue for subspecialty training after post-graduate qualification in general orthopaedics. Section 3 was on their orthopaedic training experience in post-graduate period. Questions asked about the role of female mentor, reasons of choosing orthopaedic as their career and barriers that hinder others from joining orthopaedics. Section 4 explored their current experience as orthopaedic surgeon. Questions asked about gender discrimination in their career advancement, daily work, sexual harassment in workplace and positive aspects in their career as a female. The format of this questionnaire includes open-ended, multiple choice and Likert-scale 1-10 questions. The questionnaire has been validated prior to commencement of this study. This study was approved by the Ethics Committee for Research involving Human Subjects of University Putra Malaysia.

## Results

One hundred and one (101) registered surgeons were contacted, and 82 responses were received. Response rate was 81.2%. Respondents’ demographics and employment were presented in Table I.

**Table I:** Demographics of female orthopaedic surgeons in Malaysia.

Demographics	N(%)
Ethnicity	
Malay	52 (63)
Chinese	21 (26)
Indians	5 (6)
Others	4 (5)
Age group (years)	
<36	10 (12)
36-40	39 (48)
41-45	15 (18)
46-50	9 (11)
>50	9 (11)
Marital Status	
Married	50 (61)
Not Married	28 (34)
Divorced	4 (5)
Number of child(ren)*	
0	9 (16)
1	14 (26)
2	14 (26)
3	9 (16)
≥4	8 (16)
Average child per surgeon	1.35
Age group of child(ren)**	
<7	28 (34)
7-12	26 (32)
13-17	11 (14)
≥18	8 (10)
Employment	
Ministry of Health	56 (68)
Ministry of Higher Education	17 (20)
Private Medical Institution	8 (10)
Private Education Institution	1(1)
Retired/Unemployed	1(1)
Academic title	
Professor	2 (2)
Associate Professor	3 (4)
Assistant Professor	1 (1)

*Married or divorced respondents (n=54)

**allowed >1 per married/divorced surgeon

**Fig. 1:**
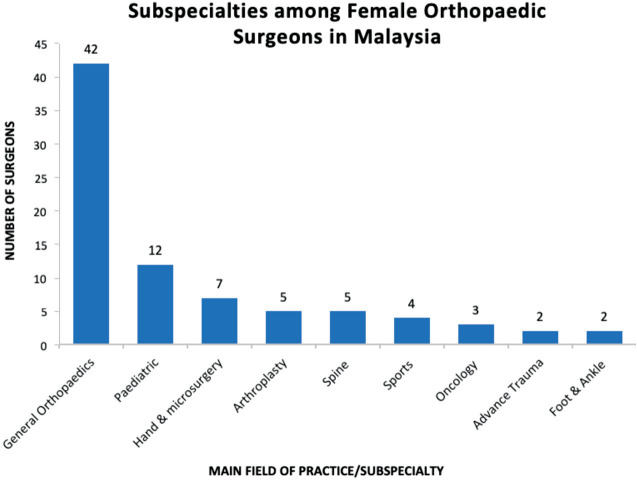
Subspecialties among female orthopaedic surgeons in Malaysia.

Forty-nine percent of female surgeons have subspecialty training, where 27% already completed training and 22% still in their training programs. Fifty-one percent do not have subspecialty training and are practicing general orthopaedics. among these surgeons in subspecialties (n=40), 30% in paediatric orthopaedic, 18% in hand and microsurgery, 12% each in arthroplasty and spine, 10% in sports surgery, 8% in oncology and 5% respectively in foot and ankle, and advance trauma (Fig. 1).

When inquiring about the reason being not trained in subspecialty (n=42), 29% expressed their interest in applying/ attempted application into subspecialty training but not being accepted yet, another 28% mentioned logistic reason, 24% were satisfied with current general orthopaedic status, 14% attributed to family commitment, another 5% were not interested and no motivation (Table II).

**Table II:** Reasons of not trained in subspecialty, gender discrimination and sexual harassment among female orthopaedic surgeons in Malaysia

Parameters	N(%)
Reason of not trained in subspecialty (n=42)*	
Interested to apply/attempt to apply (not qualified/ succeed)	17 (29)
Logistics difficulties	16 (28)
Satisfied with current status	14 (24)
Family commitment	8 (14)
Not interested	3 (5)
Gender discrimination in career (n=82)*	
No	59 (72)
Yes	23 (28)
Post graduate intake	15
Position in scientific bodies	5
Subspecialty training	4
Job promotion/opportunity in operating and learning	3
Job application	1
Grant and publication authorship	1
Interdepartmental collaboration	1
Gender discrimination and perpetrator in daily work (n=82)*	
No	33 (40)
Yes	49 (60)
Male orthopaedic colleagues	34
Patient or their family member	15
Other medical staff	15
Female orthopaedic colleagues	5
Own family/friends	3
Verbal sexual harassment (n=82)*	
No	55 (67)
Yes	27 (33)
Medical colleagues	26
Patient or their family member	4
Physical sexual harassment (n=82)*	
No	73 (89)
Yes	9 (11)
Medical colleagues	9
Patient or their family member	2

(*) respondents allowed for more than 1 option.

Seventy-nine percent of respondents agreed that they had female surgeons as their mentor in their career and 54% rate their female mentor as excellent in providing guidance and support in their career.

**Fig. 2:**
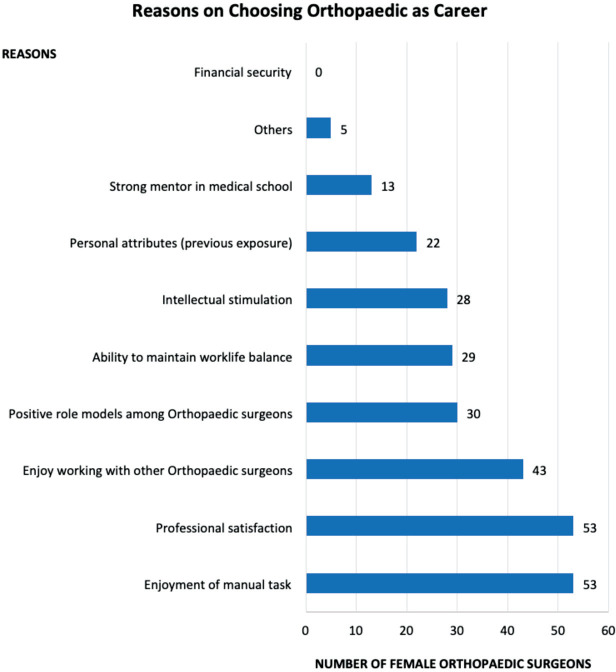
Reasons on choosing orthopaedic as career among female orthopaedic surgeons in Malaysia.

**Fig. 3:**
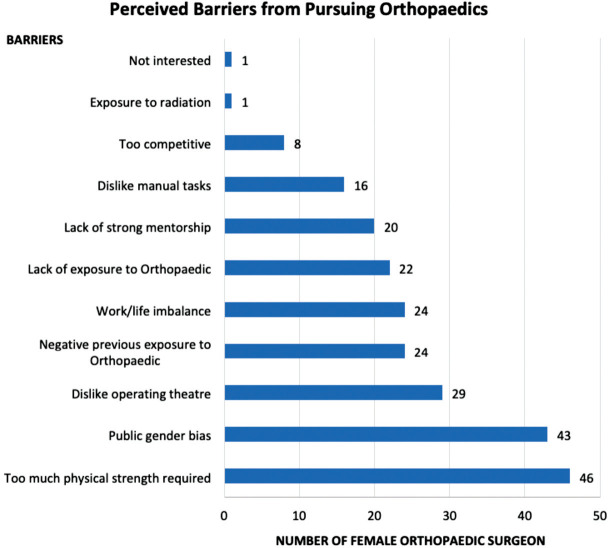
Respondents perceived barriers from pursuing orthopaedics.

When asked about the reasons on choosing orthopaedics as their career, more than 50% of respondents attribute this to enjoyment of manual tasks (64.6%), professional satisfaction (64.6%) and enjoy collaborating with other orthopaedic surgeons (52.4%). Other reasons given were positive role models among orthopaedic surgeons (36.6%), able to keep work-life balance (35.4%), intellectual satisfaction (34.1%), previous individual experiences in orthopaedics (26.8%), strong mentorship in medical school (15.9%) and others (6%) (religion/social cultural need for female orthopaedic surgeons and inspired by patients). None of the respondents took financial stability as a reason for this statement (Fig. 2). With regards to barriers for female doctors to pursue orthopaedics, the most common barriers given were too much physical strength needed for orthopaedics works (56.0%) and public gender bias in orthopaedics field (52.4%). Other barriers include dislike operating theatre (35.3%), negative earlier exposure to orthopaedics (29.3%), lack of work/life balance (29.3%), lack of exposure (26.8%), lack of mentorship (24.3%), dislike manual tasks (19.5%), orthopaedics being too competitive (9.8%), exposure to radiation in surgery and lack of interests among female doctors (1.2% each) (Fig. 3).

Seventy-two percent female surgeons denied of having any significant gender discrimination in their carrier opportunity. For the 23 surgeons (28%) who reported perceived gender discrimination, 15 reported during post graduate training intake, 5 in position in the scientific meeting and professional affiliation, 4 during subspecialty training and 3 during job promotion, opportunity during operating and learning. Others include 1each during job application, application of grant and publication authorship, and in interdepartmental collaboration (Table II).

Sixty percent of respondents reported endured discrimination for being a female orthopaedic surgeon in their daily work. For these 49 surgeons who faced discrimination, 34 from their male orthopaedic colleagues, 15 from both patients and patients’ families, 15 from other medical staffs and 5 from other female orthopaedic colleagues, 3 from their own family and friends (Table II).

Sixty-seven percent of respondents denied the experience of verbal sexual harassment. Of those 33% who had such experience, some had experienced it from more than single perpetrator. Twenty-six reported from their medical colleagues and four from their patients and patients’ family. Only 11% of the respondents reported physical sexual harassment in their entire career. Nine reported from their medical colleagues and two from patients and patients’ family (Table II).

On the other hand, 44% respondents thought they received help, tolerance, and benefits from colleagues in their career as a female surgeon and the other 56% denied receiving such benefits. Sixty percent of those who received help reported physical and manual assistance especially in the operation theatre, 40% felt they received psychosocial support.

## Discussion

Majority of the respondents were in their 30’s (12% below 36, 48% 36-40 years old) and 66% were married or divorced. The average number of children per female orthopaedic surgeon in Malaysia was only 1.35. According to data from The Department of Statistics Malaysia in 2020, fertility rate was 1.7 children per woman^4^. The reason of this comparatively low number of children among the Malaysian female orthopaedic surgeon can be due to one-third of the surgeons were not married and 16% of those married/divorced do not have a child.

Eighty-eight percent of respondents were working in government-linked institutions and 21% were in universities. For academic achievement, 28.6% of them in universities have achieved professorship. This was a noticeable achievement comparing to 13% of orthopaedic academic faculty in the United States were female professors (inclusive of assistant and associate professors)^5^.

Only 10% of female orthopaedic surgeons were working in private institution. This suggested that female surgeons were more prone to continue their career in public service which provide more stability and flexibility as compared to private sector where surgeons usually work as individual practitioners.

About half of female orthopaedic surgeons did not further their study into subspecialities. This was contrary to a study by Jurenovich *et al* which reported 94% of female orthopaedic surgeons in the United States of America (USA) had their fellowships in subspecialties^6^. Among the subspecialty choices, paediatric orthopaedic and hand and microsurgery were the most favourable, among others. This was similar to Jurenovich *et al*, where hand and paediatric orthopaedic are the most favourable among female orthopaedic surgeons^6^. A total of 29% of respondents were keen to enter subspecialty training but were yet to be accepted. This group were the younger surgeons who have recently graduated from Master of orthopaedic surgery. Others were not interested due to logistic transfer of workplace during and after training. This was an area which can be investigated to provide a secured post in a permanent location to attract more women in subspecialty training.

Lack of mentorship was often cited as one of many obstacles aspiring female doctors when considering a career in surgery or deciding on a subspecialty^7-9^. Strong mentorship fosters professional advancement which in turn increases the representation of women in leadership roles. A report on 529 orthopaedic residents in 2013 found that significantly more female than male orthopaedic residents felt that having a role model of the same sex or ethnicity was a positive factor to enter orthopaedics^10^. We reported similar findings, 79% of respondents reported presence of female mentor during their orthopaedic training and 52% grade them as excellent in providing guides and support.

When asked about their reasons of choosing orthopaedic surgery as their career, enjoyment of manual task and professional satisfaction were the highest percentage, followed by enjoying the environment working with orthopaedic colleagues, positive role model/mentor, maintaining work-life balance, and past personal experience in orthopaedics as a patient or presence of family member in orthopaedics. A small percentage but notable reason was some being inspired by patients and feeling the need to help more female patients in religious and socio-cultural context. The reasons given were similar with other countries^6,11,12^ with exception of religious, social-cultural needs. Some female patients felt more comfortable and open to discuss their illness with female doctors, especially in orthopaedics where clinical examination and management involve deep palpation and examination of covered body area. Interestingly, none of the respondents chose orthopaedics due to financial security or higher income compared to other specialities as reported in other countries^11^. Personal interests and attributes remained the major positive influence that affect their choice of career.

The most common perceived barriers for women to pursue orthopaedics is that orthopaedic is a male dominant field, where excessive physical strength and mechanical work is required. This misconception has been reported across multiple studies in women in orthopaedics^11,13^. Instruments and tools used nowadays in surgery are biomechanically more efficient and ergonomic with the correct technique of application.

The next common barrier is public gender bias. The idea of implicit bias has been recently explored as a contributing barrier to the advancement of women in medicine and surgery^14-16^. A study conducted in 2009, showed that 13.0% of the 54 orthopaedic surgeons surveyed felt that women are incapable of operating, while 5.6% believed that women should be purposefully pressurised into leaving surgery, and 21% believed that a woman’s family responsibilities should not be accommodated in surgery^17^. In the 2017 Global Gender Gap Report, Malaysia was ranked 104 out of 144 countries surveyed, indicating that women in in Malaysia still fall behind in their social, political, and economic status compared to other countries of equivalent economic standing^18^. Our largest ethnics being Malays, Chinese and Indians. The socio-cultural background of these ethnicities is fundamentally similar in gender roles. In traditional Asian society, while the men's primary role is to engage in productive activities in the public domain, women are usually assigned a complementary role confined to the domestic space, raising the children and being a good wife^19,20^. In the eye of general public, male surgeons are looked-up upon comparing female counterpart.

Many acknowledged the existence of gender discrimination in orthopaedics but not many recognised it as an important ethical issue. Almost one third of the respondents perceived gender discrimination in carrier opportunity, and half of the respondents experienced it during the post graduate training intake and 18% during post-graduate training. Other studies reported similarly, where female candidates get questions pertaining their personal and married life more than the male candidates during the interview^21^. Interviewers’ intention was usually good, to prompt these candidates to face upcoming challenges in post-graduate training, not only of the program itself, but also to expect frequent transfer and travelling that can be difficult for a family without strong support system. On the other hand, some of the respondents felt discriminated in positions in scientific bodies, job promotion and other career opportunities. Understandably, it can be difficult for a lady to break the “glass ceiling” or even secure high position in these scientific bodies. We are proud to have four female presidents in Malaysian Orthopaedic Association history and having the first female orthopaedic surgeon to hold the president flag of ASEAN Orthopaedic Association. These few exceptional women carry considerable burden to exhibit their best selves as they will always be presumed as a representative for entire female group. Once their role and leadership were accepted by the society, it will be a lot easier for the younger generation of female surgeons to follow their path in the future.

On a further note, 60% of respondents reported discrimination for being a female orthopaedic surgeon with majority of them (47%, n=49) experienced it from their male orthopaedic colleagues and another 7% experienced it from female colleagues. There is a pressing need that these female surgeons to show their utmost commitment and present their best skill to prove themselves. Gender discrimination from patients and patients’ families, other medical staffs, even unexpectedly, from their own family and friends, were part of the societal bias.

In a study of 927 members of the American Academy of Orthopaedic Surgeons, women were significantly more likely than men to experience discrimination (84% vs 59%, p<0.05) and sexual harassment (54% vs 10%, p<0.05)^22^. Studies regarding workplace sexual harassment in Malaysia are scarce. A local survey involving 586 public workers found that 50% of women respondents reported facing some form of sexual harassment in their workplace^23^. Another research in Malaysian workplaces in volving 1483 workers revealed that 38% of women respondents had experienced one or more forms of sexual harassment^24^. It is inevitable for women in orthopaedics to encounter or perceived harassment in a male-dominant industry. The term “sexual harassment” brings controversy in its definition and carries significant social stigma, especially in Asian region. Sexual harassment has been much argued to be the perceptions of the victims, in most circumstances, under-estimated by the perpetrators^25^.

Over 58% of respondents in a study felt that gendered language exists within surgical fraternity^22^. A total of 33% of our respondents felt they were verbally harassed and 11% had experienced physical harassment. Most of the cases were from medical colleagues. Even though there are 10% of female surgeons in the fraternity, it is difficult to change the gendered language behaviour. Inappropriate comments on body parts, obscene jokes and sexist assumptions are among the common verbal harassment reported. Fortunately, no serious offence reported by female orthopaedic surgeons in Malaysia as of date. Our survey revealed lower percentage of workplace sexual harassment than other fields in Malaysia (50%, and 38% as reported by Marican, Ng and Othman)^23,24^. Orthopaedic routines are full of manual work that require several team members, some work in close vicinity, e.g., manipulating fractures, dislocation or operating in a close distance. These are the common places where physical contact happens, unconscious or purposeful inappropriate body contact, can be perceived as physical harassment. It is the responsibilities of every hospital management to educate their staff including both genders to handle this issue professionally and show their transparency in handling those cases while keeping the victim anonymous. Non-bias investigation will encourage victims to stand forefront and return their faith in hospital disciplinary system. Continuous education should be provided to impart the principle of gender equity and respect.

In our survey, being perceived as the weaker gender in working among male colleagues brings certain benefits as reported by our respondents. A total of 44% respondents highlighted that help and leeway were given to them by male colleagues at work. They received manual work assistance, tolerance of working hours, leave for childcare and family emergencies, and benefits during pregnancy. During pregnancy, female surgeons usually will be given less night duty and on-calls after seven or eight months of pregnancy, exception from doing heavy operational work and entering operation theatre that require radiation with image intensifier. Male colleagues sometimes will assist in difficult manual cases for pregnant surgeon. Our findings support that the sociocultural behaviours of Malaysian society emphasises women’s’ role as a mother and family caretaker above career importance. The male colleagues in Malaysia, compared with those in other countries, can be said to be more accommodating and understanding of the work stress on female colleagues during pregnancy and with children.

This study has several limitations that should be considered in interpreting its findings: (1) This is a cross-sectional survey, which carry possibility of recall bias and do not explain the cause and relationship in between variables. (2) This study included female surgeons who qualified across 40 years (1980-2020) and did not include those who are still in orthopaedic training or general medical officer and house officer who work in orthopaedic department and might experience different challenges and experience from their seniors. Thus, this might not be representative of all female doctors who work in orthopaedics.

This study brought new insight into the representation of female in orthopaedic from Malaysian female orthopaedic surgeons’ perspectives. By analysing their reasons of choice and barriers in pursuing orthopaedics, programmes and policies can be reinforced to be more engaging on both genders in promoting orthopaedic as a career to younger doctors and medical students. More study in this aspect can be conducted in the future to explore on various initiative to create a healthy, collaborative orthopaedic community that representing general population.

## Conclusion

The reasons for Malaysian female orthopaedic surgeons to choose orthopaedic as their career and the barriers perceived to hinder other females from choosing orthopaedics were similar to reports worldwide. Gender discrimination and sexual harassment are no exception for female orthopaedic surgeons in Malaysia. However, the incidence is much lower than reported in other industries and countries. Religious and social cultural need for female orthopaedic surgeons and the support given by male colleagues to married female surgeons marked a unique phenomenon in Malaysian culture.
